# Supplementary material for: Dietary Intake by Food Source and Eating Location in Low- and Middle-Income Chilean Preschool Children and Adolescents from Southeast Santiago
Source: Nutrients. 2019 Jul 23;11(7):1695. doi: 10.3390/nu11071695 (PMC6683044; doi:10.3390/nu11071695)
Supplement: Supplementary file 1 [file nutrients-11-01695-s001.pdf]

Supplementary Material

Table S1. Food and beverage groups used for classification

Food group name	Type of group	Food group description
Cereal-based foods	Basic	Includes grains and grain-based flours and starches, dry pasta and noodles, dough, packaged or fresh breads and bread crumbs but excludes crackers.
Breakfast cereals	Nonbasic	Includes ready-to-eat and not ready-to-eat breakfast cereals with rice, oats, corn, quinoa, and/or wheat as primary ingredients.
Grain-based desserts	Nonbasic	Includes sweet bakery products and sweet cookies, cereal and protein bars; with or without added fruits, nuts, or chocolate; made with grain as a primary or major ingredient.
Sweets and non-grain-based desserts	Nonbasic	Includes sweet products, sweet spreads, and sweet dairy-based desserts, with or without added fruit or added sugar, in any edible form, frozen or not, with no or minimal grain content.
Caloric sweeteners	Nonbasic	Includes caloric sweeteners, varying solutions of glucose or fructose.
Noncaloric sweeteners	Nonbasic	Includes noncaloric sweeteners, artificial or natural.
Salty snacks	Nonbasic	Includes crackers and salty snacks made from any grain, starchy vegetable, or tuber and includes nuts, seeds, and nut and seed butters.
Meat, poultry, and meat substitutes	Basic	Includes processed or unprocessed meat, poultry, and meat substitutes, with or without added ingredients.
Fish and seafood	Basic	Includes processed or unprocessed fish, seafood, and seaweed products.
Dairy products and dairy substitutes	Basic	Includes dairy-based food products and dairy substitutes and includes cheese, yogurt, cream, evaporated and condensed milk, soy-based yogurt, and soy-based cheese.
Eggs	Basic	Includes chicken, turkey, quail, and other animal eggs and egg substitutes; fresh, raw, or processed; and includes cooked eggs, with or without added ingredients.
Legumes	Basic	Includes fresh or processed legumes, beans, lentils, and peas, with or without added ingredients, and includes soy beans but not soy-based products.
Fruits, vegetables, and mushrooms	Basic	Includes processed and unprocessed fruits, vegetables, and mushrooms, with or without added ingredients.
Condiments and sauces	Nonbasic	Includes sauces or other preparations used to enhance or add flavor to foods.
Salt and seasonings	Nonbasic	Includes salt, salt substitutes, spices, spice mixes, and seasonings.
Soups	Nonbasic	Includes dry, instant, canned, and prepared soups and includes bouillon, stock, and broth.
Oils and fats	Basic	Includes oils and fats derived from plants, seeds, or animal sources and includes butter, lard, and margarine.
Mixed dishes	Basic	Includes dishes and other food items containing mixtures or combinations of vegetables, legumes, meats, poultry, fish, seafood, dough, bread, grains, and seasonings prepared or cooked in any form; may be packaged or unpackaged, may be ready to eat, or may require preparation.
Empanadas and sandwiches	Nonbasic	Bread or pastry items with savory fillings or toppings; may be packaged or unpackaged, may be ready to eat, or may require preparation.
Fast foods	Nonbasic	Includes fast food items purchased as single items or as meal combinations, such as burgers, pizzas, chicken, fries, combo meal packages, and desserts.
Baby foods	Basic	Includes baby food products, pureed, with cereals, fruits, vegetables, or meats, and includes *picados* and *colados.*
**Beverage group name**	**Type of group**	**Beverage group description**
Sugar-sweetened beverages (SSBs)	Nonbasic	Includes industrialized flavored waters, sport drinks, energy drinks, sodas, and fruit and vegetable juices, milk, dairy drinks, drinkable yogurt, fermented milk, soy-based beverages, other dairy substitutes, and milk modifiers with sugar.
Water	Basic	Includes plain water and industrialized flavored waters without sugar.
Diet sport and energy drinks	Nonbasic	Includes sport drinks and energy drinks without sugar.
Sodas without added sugar	Nonbasic	Includes sodas without sugar.
Diet industrialized fruit and vegetable juices	Nonbasic	Includes industrialized fruit and vegetable juices without sugar.
Unsweetened dairy drinks and dairy substitutes	Basic	Includes milk, dairy drinks, drinkable yogurt, fermented milk, soy-based beverages, other dairy substitutes, and milk modifiers that are plain or flavored without sugar.
100% fruit and vegetable juices	Nonbasic	Includes fruit and vegetable juices with no added ingredients.
Coffee and tea	Nonbasic	Includes instant, powder or granule, roasted, or ground coffee beverages; drinks containing coffee with other ingredients, such as milk or sugar; and coffee substitutes of plant origin, such as soy, chicory, or barley; and includes regular and herbal teas made from plant parts in all forms; such as leaves, powder, or prepared tea bags; to brew or ready to drink.
Special formulas and nutritional supplements	Basic	Includes infant, toddler, maternal, and elderly special milks and nutritional supplements intended to meet nutritional requirements for an age or stage of life.

Table S2. Per capita and per consumer intake of calories and key nutrients of concern by food source* among low- and middle-income Chilean preschool children and adolescents, 2016

Preschool children (n = 839)						Adolescents (n = 643)					
**Food source**	% of total daily intake	Per capita		Per consumers		**Food source**	% of total daily intake	Per capita		Per consumers	
		Mean	SE†	Mean	SE			Mean	SE	Mean	SE
**Home (n = 839)**						**Home (n = 641)**					
**Energy, kcal/day**	82.9%	1,041.6	14.2	1,041.6	14.2	**Energy, kcal/day**	77.6%	1,416.5	25.5	1,420.9	25.4
**Saturated fats, g/day**	82.9%	11.5	0.2	11.5	0.2	**Saturated fats, g/day**	77.2%	15.4	0.4	15.5	0.4
**Total sugars, g/day**	86.8%	77.3	1.3	77.3	1.3	**Total sugars, g/day**	78.3%	78.3	2.0	78.5	2.0
**Sodium, mg/day**	86.2%	1,284.9	24.0	1,284.9	24.0	**Sodium, mg/day**	80.7%	1,887.2	40.5	1,893.1	40.4
**Away from home (n = 497)**						**Away from home (n = 468)**					
**Energy, kcal/day**	17.1%	215.6	10.1	364.0	13.4	**Energy, kcal/day**	22.4%	409.8	17.4	563.0	19.7
**Saturated fats, g/day**	17.0%	2.4	0.1	4.0	0.2	**Saturated fats, g/day**	22.8%	4.6	0.2	6.3	0.3
**Total sugars, g/day**	13.2%	11.7	0.6	19.8	0.9	**Total sugars, g/day**	21.7%	21.7	1.2	29.8	1.4
**Sodium, mg/day**	13.8%	206.2	10.6	348.1	14.9	**Sodium, mg/day**	19.3%	451.8	24.4	620.7	29.9

*We determined the food source by combining foods purchased at groceries, convenience stores, or supermarkets and products made at home into one category (home) and combining ready-to-eat food obtained at school and other sources, such as restaurants and fast food establishments, into another category (away from home).
† SE, Standard error.

Table S3. Per capita and per consumer intake of calories and key nutrients of concern by eating location* among low- and middle-income Chilean preschool children and adolescents, 2016

Preschool children (n = 839)						Adolescents (n = 643)					
**Eating location**	% of total daily intake	Per capita		Per consumers		**Eating location**	% of total daily intake	Per capita		Per consumers	
		Mean	SE†	Mean	SE			Mean	SE	Mean	SE
**Home (n = 831)**						**Home (n = 632)**					
**Energy, kcal/day**	66.3%	834.0	13.9	842.0	13.8	**Energy, kcal/day**	62.8%	1,146.9	24.5	1,166.9	24.1
**Saturated fats, g/day**	68.8%	9.6	0.2	9.7	0.2	**Saturated fats, g/day**	59.6%	11.9	0.4	12.1	0.4
**Total sugars, g/day**	62.3%	55.4	1.1	56.0	1.1	**Total sugars, g/day**	58.5%	58.5	1.8	59.5	1.8
**Sodium, mg/day**	73.6%	1,097.2	23.3	1,107.7	23.2	**Sodium, mg/day**	69.0%	1,613.5	40.4	1,641.6	40.2
**School (n = 616)**						**School (n = 496)**					
**Energy, kcal/day**	22.3%	280.5	9.9	382.1	10.8	**Energy, kcal/day**	26.6%	486.7	16.7	630.9	16.8
**Saturated fats, g/day**	19.2%	2.7	0.1	3.6	0.1	**Saturated fats, g/day**	28.9%	5.8	0.2	7.5	0.2
**Total sugars, g/day**	25.6%	22.8	0.7	31.0	0.8	**Total sugars, g/day**	30.1%	30.1	1.2	39.0	1.3
**Sodium, mg/day**	15.5%	230.9	9.6	314.5	11.3	**Sodium, mg/day**	20.5%	480.4	20.3	622.8	22.6
**Other (n = 384)**						**Other (n = 229)**					
**Energy, kcal/day**	11.3%	142.7	9.8	311.8	18.0	**Energy, kcal/day**	10.6%	192.7	16.5	541.0	36.3
**Saturated fats, g/day**	12.0%	1.7	0.1	3.6	0.2	**Saturated fats, g/day**	11.5%	2.3	0.2	6.5	0.5
**Total sugars, g/day**	12.1%	10.8	0.8	23.6	1.4	**Total sugars, g/day**	11.4%	11.4	1.0	32.1	2.3
**Sodium, mg/day**	10.9%	163.1	13.7	356.3	26.7	**Sodium, mg/day**	10.5%	245.0	24.2	687.9	57.2

*We designated the eating location as home if food was consumed at home, school if food was consumed at school, and other if food was consumed at another person's home, at a food court, at a cinema, at a restaurant, in the street, on transportation, or at another location.
† SE, Standard error.

Table S4. Per capita and per consumer intake of calories and key nutrients of concern by food source* among low- and middle-income Chilean preschool children and adolescents, 2016, excluding outliers for saturated fats and sodium

**Preschool children (n = 831)**						**Adolescents (n = 637)**					
**Food source**	% of total daily intake	Per capita Mean	Per capita SE†	Per consumers Mean	Per consumers SE	**Food source**	% of total daily intake	Per capita Mean	Per capita SE	Per consumers Mean	Per consumers SE
**Home (n = 831)**						**Home (n = 635)**					
**Energy, kcal/day**	82.8%	1,034.7	13.8	1,034.7	13.8	**Energy, kcal/day**	77.6%	1,408.4	25.5	1,412.8	25.4
**Saturated fats, g/day**	83.1%	11.4	0.2	11.4	0.2	**Saturated fats, g/day**	77.0%	15.2	0.4	15.2	0.4
**Total sugars, g/day**	86.9%	76.8	1.2	76.8	1.2	**Total sugars, g/day**	78.4%	78.0	2.0	78.3	2.0
**Sodium, mg/day**	86.1%	1,267.8	22.6	1,267.8	22.6	**Sodium, mg/day**	80.6%	1,859.8	38.6	1,865.6	38.5
**Away from home (n = 492)**						**Away from home (n = 464)**					
**Energy, kcal/day**	17.2%	214.5	10.0	362.3	13.3	**Energy, kcal/day**	22.4%	407.4	17.3	559.3	19.5
**Saturated fats, g/day**	16.9%	2.3	0.1	3.9	0.2	**Saturated fats, g/day**	23.0%	4.5	0.2	6.2	0.3
**Total sugars, g/day**	13.1%	11.6	0.6	19.6	0.9	**Total sugars, g/day**	21.6%	21.5	1.2	29.5	1.4
**Sodium, mg/day**	13.9%	205.5	10.6	347.1	14.8	**Sodium, mg/day**	19.4%	447.0	24.2	613.7	29.7

*We determined the food source by combining foods purchased at groceries, convenience stores, or supermarkets and products made at home into one category (home) and combining ready-to-eat food obtained at school and other sources, such as restaurants and fast food establishments, into another category (away from home).

† SE, Standard error.

Table S5. Per capita and per consumer intake of calories and key nutrients of concern by eating location* among low- and middle-income Chilean preschool children and adolescents, 2016, excluding outliers for saturated fats and sodium

**Preschool children (n = 831)**						**Adolescents (n = 637)**					
**Eating location**	% of total daily intake	Per capita Mean	Per capita SE†	Per consumers Mean	Per consumers SE	**Eating location**	% of total daily intake	Per capita Mean	Per capita SE	Per consumers Mean	Per consumers SE
**Home (n = 823)**						**Home (n = 626)**					
**Energy, kcal/day**	66.2%	826.4	13.6	834.4	13.4	**Energy, kcal/day**	62.8%	1,139.8	24.4	1,159.8	24.1
**Saturated fats, g/day**	68.5%	9.4	0.2	9.5	0.2	**Saturated fats, g/day**	59.2%	11.7	0.3	11.9	0.3
**Total sugars, g/day**	62.0%	54.9	1.1	55.4	1.1	**Total sugars, g/day**	58.5%	58.2	1.7	59.2	1.8
**Sodium, mg/day**	73.5%	1,083.2	22.2	1,093.7	22.1	**Sodium, mg/day**	68.9%	1,589.6	38.5	1,617.6	38.2
**School (n = 611)**						**School (n = 493)**					
**Energy, kcal/day**	22.5%	281.2	9.9	382.5	10.9	**Energy, kcal/day**	26.9%	488.2	16.7	630.8	16.9
**Saturated fats, g/day**	19.5%	2.7	0.1	3.7	0.1	**Saturated fats, g/day**	29.5%	5.8	0.2	7.5	0.2
**Total sugars, g/day**	25.8%	22.8	0.7	31.0	0.8	**Total sugars, g/day**	30.3%	30.2	1.2	39.0	1.3
**Sodium, mg/day**	15.7%	231.8	9.6	315.2	11.3	**Sodium, mg/day**	20.8%	479.7	20.3	619.8	22.6
**Other (n = 376)**						**Other (n = 224)**					
**Energy, kcal/day**	11.3%	141.5	9.9	312.8	18.3	**Energy, kcal/day**	10.3%	187.8	16.2	534.1	36.1
**Saturated fats, g/day**	11.9%	1.6	0.1	3.6	0.2	**Saturated fats, g/day**	11.4%	2.2	0.2	6.4	0.5
**Total sugars, g/day**	12.2%	10.8	0.8	23.8	1.4	**Total sugars, g/day**	11.2%	11.1	1.0	31.7	2.3
**Sodium, mg/day**	10.7%	158.4	13.0	350.0	25.4	**Sodium, mg/day**	10.3%	237.5	23.6	675.4	56.6

*We designated the eating location as home if food was consumed at home, school if food was consumed at school, and other if food was consumed at another person's home, at a food court, at a cinema, at a restaurant, in the street, on transportation, or at another location.

† SE, Standard error.

Table S6. Per capita and per consumer intake of calories and key nutrients of concern by food source* among low- and middle-income Chilean preschool children and adolescents during weekends, 2016.

Preschool children (n = 172)						Adolescents (n = 168)					
**Food source**	% of total daily intake	Per capita Mean	Per capita SE†	Per consumers Mean	Per consumers SE	Food source	% of total daily intake	Per capita Mean	Per capita SE	Per consumers Mean	Per consumers SE
**Home (n = 172)**						**Home (n = 168)**					
**Energy, kcal/day**	86.3%	996.1	27.9	996.1	27.9	**Energy, kcal/day**	84.3%	1,625.6	49.9	1,625.6	49.9
**Saturated fats, g/day**	84.8%	11.2	0.5	11.2	0.5	**Saturated fats, g/day**	82.5%	17.9	0.7	17.9	0.7
**Total sugars, g/day**	85.5%	70.2	2.7	70.2	2.7	**Total sugars, g/day**	82.7%	84.6	3.9	84.6	3.9
**Sodium, mg/day**	85.4%	1,296.5	47.5	1,296.5	47.5	**Sodium, mg/day**	85.7%	2,278.0	85.7	2,278.0	85.7
**Away from home (n = 87)**						**Away from home (n = 81)**					
**Energy, kcal/day**	13.7%	158.6	19.6	313.6	30.7	**Energy, kcal/day**	15.7%	302.2	44.0	626.9	76.5
**Saturated fats, g/day**	15.2%	2.0	0.3	4.0	0.5	**Saturated fats, g/day**	17.5%	3.8	0.6	7.8	1.1
**Total sugars, g/day**	14.5%	11.9	1.8	23.5	3.1	**Total sugars, g/day**	17.3%	17.7	3.1	36.7	5.8
**Sodium, mg/day**	14.6%	221.6	33.9	438.2	58.5	**Sodium, mg/day**	14.3%	380.5	59.5	789.3	106.2

*We determined the food source by combining foods purchased at groceries, convenience stores, or supermarkets and products made at home into one category (home) and combining ready-to-eat food obtained at school and other sources, such as restaurants and fast food establishments, into another category (away from home).

† SE, Standard error.

Table S7. Per capita and per consumer intake of calories and key nutrients of concern by eating location* among low- and middle-income Chilean preschool children and adolescents during weekends, 2016

Preschool children (n = 172)						Adolescents (n = 168)					
**Eating location**	% of total daily intake	Per capita Mean	Per capita SE†	Per consumers Mean	Per consumers SE	**Eating location**	% of total daily intake	Per capita Mean	Per capita SE	Per consumers Mean	Per consumers SE
**Home (n = 164)**						**Home (n = 164)**					
**Energy, kcal/day**	72.8%	840.0	33.3	881.0	31.5	**Energy, kcal/day**	71.2%	1,372.6	56.1	1,406.1	54.9
**Saturated fats, g/day**	72.4%	9.6	0.5	10.0	0.5	**Saturated fats, g/day**	68.7%	14.9	0.7	15.2	0.7
**Total sugars, g/day**	73.3%	60.2	2.9	63.1	2.8	**Total sugars, g/day**	67.7%	69.3	3.9	71.0	3.9
**Sodium, mg/day**	71.6%	1,086.7	50.4	1,139.7	49.3	**Sodium, mg/day**	74.0%	1,968.3	91.6	2,016.3	90.7
**School (n = 2)**						**School (n = 7)**					
**Energy, kcal/day**	0.7%	7.6	6.2	655.1	369.4	**Energy, kcal/day**	0.6%	11.5	5.8	277.0	101.3
**Saturated fats, g/day**	0.6%	0.1	0.1	7.0	5.5	**Saturated fats, g/day**	0.6%	0.1	0.1	3.2	1.2
**Total sugars, g/day**	0.7%	0.6	0.5	50.4	28.6	**Total sugars, g/day**	0.8%	0.8	0.4	20.4	5.5
**Sodium, mg/day**	1.5%	22.1	19.0	1,902.9	1,320.7	**Sodium, mg/day**	0.3%	8.0	5.0	191.1	105.5
**Other (n = 100)**						**Other (n = 90)**					
**Energy, kcal/day**	26.6%	307.0	33.2	528.1	45.8	**Energy, kcal/day**	28.2%	543.7	61.4	1,015.0	88.5
**Saturated fats, g/day**	27.0%	3.6	0.5	6.2	0.7	**Saturated fats, g/day**	30.7%	6.6	0.8	12.4	1.2
**Total sugars, g/day**	26.0%	21.3	2.4	36.7	3.4	**Total sugars, g/day**	31.5%	32.2	4.1	60.1	6.3
**Sodium, mg/day**	27.0%	409.3	48.0	704.0	69.0	**Sodium, mg/day**	25.7%	682.2	84.2	1,273.5	128.0

*We designated the eating location as home if food was consumed at home, school if food was consumed at school, and other if food was consumed at another person’s home, at a food court, at a cinema, at a restaurant, in the street, on transportation, or at another location.

† SE, Standard error.
